# Visual Acuity Thresholds in Preterm Newborns: An Experimental Study

**DOI:** 10.3390/children11091049

**Published:** 2024-08-28

**Authors:** Ruth Batista Bezerra Fagundes, Pedro Ykaro Fialho Silva, Mirella Telles Salgueiro Barboni, Gentil Gomes da Fonseca Filho, Valeria Azevedo de Almeida, Ingrid Guerra Azevedo, Silvana Alves Pereira

**Affiliations:** 1 Physiotherapy Departament, Faculdade Ciências da Saúde do Trairi, Universidade Federal do Rio Grande do Norte (UFRN), Santa Cruz 59200-000, Brazil; ruthbezerrafisio@gmail.com (R.B.B.F.); gentil.fonseca@ufrn.br (G.G.d.F.F.); silvana.alves@ufrn.br (S.A.P.); 2 Physiotherapy Department, Campus Universitário Lagoa Nova, Universidade Federal do Rio Grande do Norte (UFRN), Natal 59078-900, Brazil; 20221783@sarah.br (P.Y.F.S.);; 3 Department of Experimental Psychology, Universidade de São Paulo (USP), São Paulo 05508-030, Brazil; mirella.barboni@semmelweis.hu; 4 Department of Ophthalmology, Semmelweis University, 1085 Budapest, Hungary; 5 Vicerrectoría Académica, Universidad Católica de Temuco, Rudecindo Ortega 02950, Campus San Juan Pablo II, Temuco 4780000, La Araucania, Chile

**Keywords:** premature, intensive care units, visual acuity, phototherapy

## Abstract

**Purpose:** Visual acuity plays a role in mediating neurological development in infants by enabling the differentiation of shapes and discriminating objects. Given the rapid structural development of the brain in the first days of life, this aspect is particularly significant for preterm infants, who typically experience this developmental phase while hospitalized in the neonatal intensive care unit (NICU). Therefore, this study aimed to assess visual acuity thresholds in preterm infants during hospitalization and to evaluate possible correlations between visual acuity and clinical parameters. **Methods**: A cross-sectional study was conducted in an NICU in Northeast Brazil. The visual acuity thresholds were tested using the Teller Acuity Cards II, comprised of 17 gray cards, with one 4 mm diameter peephole at the center and presented with about 35% reflectance. Preterm infants were positioned supine, at 30° elevation on the laps of their caregivers. The evaluator presented both sides of the cards and observed the eye fixation and reactions on both sides. **Results:** A total of 42 preterm infants with corrected gestational age between 30 to 36 weeks and 6 days were included. Visual acuity ranged from 0.23 to 0.64 cycle per degree. The mean visual acuity threshold was 0.32 cycles per degree for preterm infants at around 32 weeks of corrected gestational age. The visual acuity was not correlated with gestational age (*p* = 0.18), and neither were birth weight (*p* = 0.83) or duration of respiratory support (*p* = 0.98). However, days of phototherapy were inversely correlated with visual acuity (*p* = 0.04). **Conclusions:** Despite the challenges of hospitalization, it was possible to carry out a psychophysical test to assess visual acuity in preterm infants. The visual acuity showed no correlation with clinical parameters such as gestational age, birth weight, and duration of respiratory support. However, there was an inverse correlation between the number of days in phototherapy and visual acuity. Understanding the visual acuity levels in preterm infants during their NICU stay can contribute to tailoring interventions and care strategies that specifically address their visual developmental needs. This knowledge may guide healthcare professionals in optimizing the NICU environment to provide appropriate visual stimuli that support neurological development.

## 1. Introduction

Visual acuity plays a crucial role in mediating neurological development in infants as it enables sharp vision, allowing them to differentiate shapes and discriminate objects [1]. Although some studies have shown that preterm newborns develop visual acuity earlier than term newborns in the first year of life [2,3], little is known about visual acuity thresholds during the first days of visual experience.

The brain shows fast structural development during the first days of life [4]. In preterm newborns, this generally occurs during hospitalization in the neonatal intensive care unit (NICU) [5]. Despite the vital support provided in the NICU, physiological factors or intense exposure to environmental lights and stimuli may affect the newborns’ development [5,6]. Additionally, longer NICU hospitalization may impair neuromotor and cognitive functions [6].

Several studies have reported assessments of neurological and visual functions in newborn infants [7,8,9,10,11]. Some assessment methods have been adapted and validated specifically for premature infants [12]. These assessments are crucial in identifying and addressing potential developmental issues early on, which is particularly important given the challenges preterm newborns face during their critical early stages of development.

Given that visual anomalies are common among young children, particularly in preterm neonates, screening for the early diagnosis and correction of visual deficiencies is crucial [13]. Abnormal visual acuity can lead to visual development abnormalities, which may result in permanent visual impairment if left untreated [13,14]. Considering the prolonged neonatal hospitalization of these premature infants, detailed knowledge of visual acuity thresholds in the hospital is essential. This information can be the first step in a long journey and help healthcare professionals establish appropriate sensory stimulation and rehabilitation programs during the critical period of visual development when cortical formation and maturation are determined [15]. This study assessed visual acuity thresholds in preterm newborns during neonatal hospitalization and evaluated possible correlations between visual acuity and clinical parameters.

## 2. Materials and Methods

### 2.1. Study Design

This cross-sectional study was conducted in the NICU of a maternity school in the northeastern region of Brazil and it was approved by the research ethics committee of the Federal University of Rio Grande do Norte (no. 1.876.007/2018). The study was performed according to resolution 466/12 of the National Health Council and the Declaration of Helsinki.

### 2.2. Sample

The study included preterm newborns born at less than 36 weeks and six days of gestation, according to the first ultrasound, who were admitted to the NICU and not under mechanical ventilation or medication (sedative or central nervous system depressants). Preterm newborns with neurological damage, such as hydrocephalus or periventricular leukomalacia, retinopathy of prematurity or congenital malformations were not invited to participate in the study. Newborns who could not complete the test due to a drop in alertness (drowsiness or crying) were excluded from the data analysis. All caregivers included were informed about the study’s objectives and provided written consent before any tests were conducted.

### 2.3. Visual Acuity Assessment

**Figure 1:**
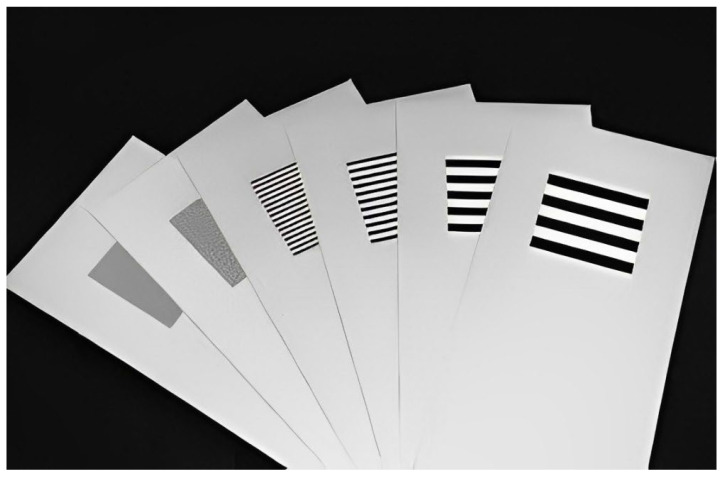
Teller Acuity Cards II.

Visual acuity thresholds were psychophysically tested using the Teller Acuity Cards II (TAC II) [16]—Figure 1. TAC II is a set of standardized tools used to assess visual acuity in infants and young children, including newborns. Each card features a rectangular patch with high-contrast black-and-white stripes (gratings) on one side and a uniform gray field on the other. The spatial frequency of the stripes varies across different cards, representing different levels of visual acuity, and is measured in cycles per degree. During the test, the examiner presents the cards one at a time to the infant, holding the card in front of the child at a fixed distance. The examiner observes the infant’s eye movements and gaze preferences, noting whether the infant shows a preference for looking at the side of the card with the stripes, which indicates that the infant can detect the contrast. By determining the finest stripe pattern that the infant can reliably detect, the examiner can estimate the infant’s visual acuity. This method is particularly useful for assessing visual function in newborns who are too young to verbally communicate or follow more complex instructions [16].

For this study, during the visual assessment, the NICU luminance was controlled at 170 (±32.92) lux, and the NICU noise ranged from 29 to 46 decibels. The noise was measured with a decibel meter connected to a mobile phone application, the Sound Meter app, and the luminance was measured using a luxmeter, Minipa MLM-1010, São Paulo, Brazil.

Preterm newborns were positioned supine (30° elevation) on the laps of their caregivers. Cards were presented at a standard distance of 19 cm, according to the TAC II manual of instructions [16]. The evaluator presented both sides of the cards using the Davida Teller method [16] and observed the eye fixation and reactions (eye or head movements or both) on both sides.

One expert examiner working at the high-risk infant care unit performed the examinations. The complete set of TAC II comprised 17 cards (25 per 5 cm × 55 per 5 cm) with one 4 mm diameter peephole at the center. The cards were gray, and they were presented with about 35% of reflectance.

Clinical parameters including gender, birth weight, weight classification, gestational age, transfontanelar ultrasound, duration of respiratory support, bronchodysplasia, sepsis, visual experience, and Apgar were evaluated through medical records to associate them with visual acuity values.

### 2.4. Statistical Analysis

Data were analyzed using SPSS (IBM Corp, Chicago, IL, USA, version 20.0). They are presented as mean and standard deviation, medians and interquartile ranges, and relative/absolute frequencies. The Kruskal–Wallis test was applied to compare non-parametric variables with visual acuity thresholds (gestational age, birth weight, duration of respiratory support, visual experience, Apgar). Spearman’s test was used to verify correlations between visual acuity thresholds and clinical parameters. The following classification was used for the degrees of correlation—weak r < 0.4; moderate 0.4 < r < 0.7 and strong when r > 0.7 [17]. To determine the limit of visual acuity for premature infants, the receiver operating characteristic (ROC) curve was calculated. Significance was set at *p* < 0.05, and 95% confidence intervals were adopted for all analyses.

## 3. Results

A total of 167 preterm newborns were admitted to the NICU during the study period; 125 did not meet the inclusion criteria. Fifty-nine were assessed, and seventeen did not complete the test because of decreased alertness (drowsiness or crying). The final sample was 42 preterm newborns (22 female); 23 were born by cesarean delivery (Table 1).

**Figure 2:**
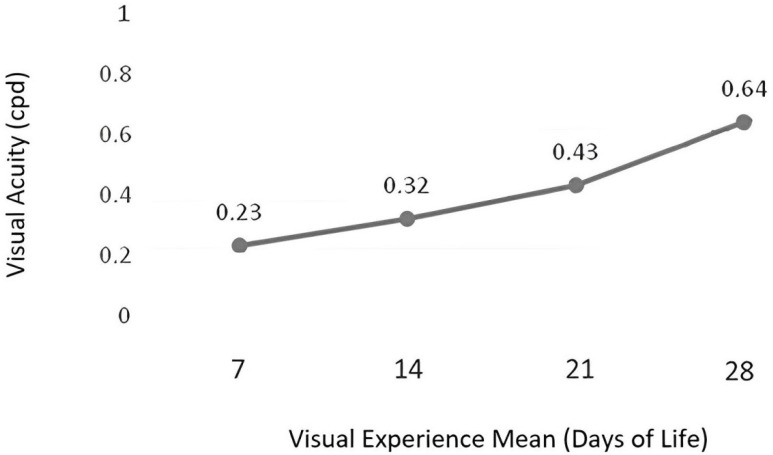
Visual experience (days of life) × visual acuity (cycle per degree—cpd).

Visual acuity ranged from 0.23 to 0.64 cycle per degree. The mean visual acuity threshold was 0.32 cycle per degree (ROC curve: *p* = 0.03, area under the curve of 0.82) for preterm infants with 31 weeks and 5 days of corrected gestational age. Greater visual experience correlates with higher visual acuity values (*p* = 0.04)—Figure 2.

Visual acuity was not correlated with corrected gestational age (*p* = 0.18), birth weight (*p* = 0.83) or duration of respiratory support (*p* = 0.98). However, days of phototherapy was inversely correlated with visual acuity (r = −0.309). The more days in phototherapy, the lower the visual acuity threshold (*p* = 0.04).

## 4. Discussion

The application of TAC II in preterm newborns showed average visual acuity thresholds of 0.32 cycles per degree in subjects around 32 weeks of corrected gestational age. Interestingly, the results indicated that visual acuity thresholds were inversely correlated with the number of days on phototherapy.

Currently, only one study has reported visual acuity in very young preterm newborns with ages ranging from 32 to 33 weeks. The authors reported that 95% of the newborns were able to discriminate TAC II stimuli between 0.64 and 0.82 cycles per degree [18]. However, the authors did not consider the chronological age or the visual experience of the newborns, hindering comparisons with the present data.

It has been shown that a threshold of 0.41 cycles per degree in 8 weeks of life in full-term newborns is sufficient to recognize the mother’s face [19,20]. De Heerring et al. [19] observed that a visual acuity of 0.5 cycles per degree was sufficient for full-term newborns to recognize faces after two days of life. The present results established an unpublished average threshold for preterm newborns between 30 to 36 weeks and six days of corrected gestational age, filling a gap in the literature.

The early recognition of an altered visual acuity in preterm newborns may allow healthcare professionals to provide adequate visual stimulation and satisfactory visual experiences required for proper visual development during early childhood [15,21,22]. As gestures and social behaviors are mainly learned via visual feedback [23], an altered visual acuity may modify the extraction of this important information from the environment [24,25,26], hindering physical and cognitive development, activities of daily life [27,28], social interaction [29], playing [30], and academic activities [31,32].

A previous study of visual acuity using visual evoked potential and behavioral measurements has found similar results in preterm and full-term newborns [33]. The authors suggested that early environmental stimulation in preterm newborns induces the maturation of the visual–motor cortical circuits, reflecting greater visual acuity in preterm newborns compared to term visual acuity. This concept may help to explain some of our findings, as we found no correlation between visual acuity and clinical parameters such as gestational age, birth weight, and duration of respiratory support. However, we observed that a greater number of days in phototherapy was associated with a lower visual acuity. These results suggest that other factors, such as visual experience, might play a more significant role in influencing visual acuity in preterm newborns.

Visual experience is a fundamental aspect in determining the development of visual acuity [22,34,35]. Since phototherapy affects visual acuity, newborns’ eyes are covered during phototherapy to prevent damage to the retina from ultraviolet radiation [36,37]. This situation deprives newborns of visual stimuli that normally exist in the environment. Previous studies evaluating early visual deprivation in newborns with congenital cataracts have demonstrated that visual deprivation in the first days of life affects the development of several functions of the visual system [38]. Therefore, visual experience in the first weeks of life plays a crucial role in the formation and maturation of neural circuits, which are essential for the proper development of visual functions [39,40].

The mean luminance (170 lux) measured in the ambient setting was lower than expected in the NICU [40]. The NICU is usually brighter, and about 30% of the light is delivered to the eyelid of newborns, decreasing their opening frequency and impairing the physiological stability and organization of cortical circuits [41]. High luminance levels are associated with low weight gain and behavioral and sleep disturbances [42]. In addition, the NICU environment may modulate not only visual acuity, but also color vision, attention, habituation, and visual regulation.

Although noise and luminance were controlled during the examinations, the trajectory of visual acuity at different ages was not assessed after hospitalization, limiting the interpretation of these findings. Another limitation, and a future perspective, is the lack of follow-up assessments at older ages such as 3, 6, and 12 months. Evaluating visual acuity at these later stages would provide a more comprehensive understanding of visual development and the long-term effects of early interventions.

Hospitalization exposes PT to clinical procedures that generate noise [43] and excessive luminance [42] and may trigger functional and microstructural changes. Therefore, investigating visual acuity during the neonatal period may be useful to investigate compensatory mechanisms after brain injury.

Despite the challenges of hospitalization, it was possible to carry out a psychophysical test to assess visual acuity in preterm infants. The visual acuity showed no correlation with clinical parameters such as gestational age, birth weight and duration of respiratory support. However, the more days in phototherapy, the lower the visual acuity. Understanding the visual acuity levels in preterm infants during their NICU stay can contribute to tailoring interventions and care strategies that specifically address their visual developmental needs. This knowledge may guide healthcare professionals in optimizing the NICU environment to provide appropriate visual stimuli that support neurological development.

## Figures and Tables

**Table 1:** Sample characteristics.

Variables	Mean (SD)/Median (Q1–Q3) n = 42	
Gestational age at birth (weeks)	30 (±2.23) ^†^	
Corrected gestational age (weeks)	32 (30–36)	
Visual experience (days) *	9 (1–57)	
Weight (grams)	1290.20 (±267.10) ^†^	
Apgar (first minute)	8 (4–9)	
Apgar (fifth minute)	9 (5–9)	
	**Frequency (% and N)**	
Type of delivery **	Vaginal	37.80% (14)
	Cesarean	62.20% (23)
Gender	Male	47.60% (20)
	Female	52.40% (22)

* Number of days after birth. ** For this variable there are missing data, totaling 37 newborns. ^†^ Mean and standard deviation.

## Data Availability

The raw data supporting the conclusions of this article will be made available by the authors on request.
